# Impacts of Cropping Systems on Aggregates Associated Organic Carbon and Nitrogen in a Semiarid Highland Agroecosystem

**DOI:** 10.1371/journal.pone.0165018

**Published:** 2016-10-20

**Authors:** Jiashu Chu, Tianzhe Zhang, Weidong Chang, Dan Zhang, Saman Zulfiqar, Aigen Fu, Yaqi Hao

**Affiliations:** Key Laboratory of Resource Biology and Biotechnology in Western China (Northwest University), Ministry of Education, Xi’an, Shaanxi, 710069, China; Tennessee State University, UNITED STATES

## Abstract

The effect of cropping system on the distribution of organic carbon (OC) and nitrogen (N) in soil aggregates has not been well addressed, which is important for understanding the sequestration of OC and N in agricultural soils. We analyzed the distribution of OC and N associated with soil aggregates in three unfertilized cropping systems in a 27-year field experiment: continuously cropped alfalfa, continuously cropped wheat and a legume-grain rotation. The objectives were to understand the effect of cropping system on the distribution of OC and N in aggregates and to examine the relationships between the changes in OC and N stocks in total soils and in aggregates. The cropping systems increased the stocks of OC and N in total soils (0–40 cm) at mean rates of 15.6 g OC m^-2^ yr^-1^ and 1.2 g N m^-2^ yr^-1^ relative to a fallow control. The continuous cropping of alfalfa produced the largest increases at the 0–20 cm depth. The OC and N stocks in total soils were significantly correlated with the changes in the >0.053 mm aggregates. 27-year of cropping increased OC stocks in the >0.053 mm size class of aggregates and N stocks in the >0.25 mm size class but decreased OC stocks in the <0.053 mm size class and N stocks in the <0.25 mm size class. The increases in OC and N stocks in these aggregates accounted for 99.5 and 98.7% of the total increases, respectively, in the continuous alfalfa system. The increases in the OC and N stocks associated with the >0.25 mm aggregate size class accounted for more than 97% of the total increases in the continuous wheat and the legume-grain rotation systems. These results suggested that long-term cropping has the potential to sequester OC and N in soils and that the increases in soil OC and N stocks were mainly due to increases associated with aggregates >0.053 mm.

## Introduction

Crop rotation effects on soil organic carbon (OC) and nutrients from agroecosystems and improve soil fertility, OC and nitrogen (N) contents and nutrient-use efficiencies [1–4]. Al-Kaisi et al. reported that increasing crop diversity by including perennial grasses could effectively improve C and N sequestration in Iowa soils [3]. Dwivedi et al. suggested that the inclusion of forage cowpea in a rice-wheat system could increase the use efficiencies of N and phosphorus on the Indo-Gangetic Plain of India. Acosta-Martinez et al. demonstrated that a peanut-based cropping system significantly improved the structure and functionality of the microbial community in a sandy soil [4]. Aziz et al. reported that multiple cropping system effect soil carbon and nitrogen status, and also improve soil functional properties [5–7]. Such information need to be well addressed to understand the mechanism behind the responses of soil OC and N to changes in cropping systems.

The distributions of soil aggregates and aggregate-associated OC and N are often used to indicate changes in soil structure and in OC and N stocks and to assess the stability of the OC and N in soils to changes in soil-management and cropping systems [8–11]. The effects of different tillage practices on the distribution of aggregates and aggregate-associated OC in agroecosystems have been extensively studied. For example, Paul et al demonstrated that conventional tillage reduced the stability of aggregates and aggregate-associated OC compared with reduced tillage [12]. Six et al. observed higher OC contents in aggregates in untilled soils than in conventionally tilled soils [13]. They suggested a faster turnover rate of macroaggregates and a lower stability of new OC in aggregates with conventional tillage compared with no tillage. Simpson et al. found a preferential accumulation of microbial C in aggregate structures of untilled soils compared with conventionally tilled soils [14]. Denef et al. reported significant C sequestration in microaggregates of untilled soils with drastically different clay minerals [15]. Little is known, however, about the effects of different cropping systems on soil OC and N associated with aggregates.

Most previous studies of the effect of cropping system on soil OC and N usually included fertilizer (organic or chemical) treatments, while very few studies have compared the effects of cropping systems that did not include fertilization. The application of fertilizers can significantly affect soil OC and N. For example, Jagadamma et al. found significantly higher OC and N contents with the application of N in continuously cropped corn and in corn-soybean rotation systems [16]. The effects of fertilization should thus be carefully treated and be separated from those of the cropping systems.

Fallow treatments (bare land without crops) have often been ignored, and the effect of cropping system on soil OC and N has mostly been assessed by only comparing different cropping systems [16–17]. The inclusion of a fallow treatment can provide basic information about the study site and can be used as a true control for analyzing the effects of cropping systems, because the stocks and distributions of OC and N are significantly different in fallow and cropped land. For example, Yousefi et al. found that the soil of a cropping system that included fallow land had relatively lower OC mean weights and aggregate diameters than a cropping system without fallow land [18]. The effects of fertilization should thus be assessed separately, and fallow treatments should be included to provide a more accurate understanding of how soil OC and N respond to cropping systems.

In this study, a 27-year experiment in a highland agricultural area compared the distributions of aggregate and aggregate-associated OC and N among three unfertilized cropping systems. The objectives were to understand the effect of cropping system on the distribution of OC and N in aggregates and to examine the relationships between the changes in OC and N in total soils and in aggregates.

## Materials and Methods

### Study site

The field experiment was initiated in September 1984 in Changwu County, Shaanxi Province, China (35°12′N, 107°40′E). The study site has a warm-temperate, subhumid, continental climate. During the period 1984 to 2011, the mean annual temperature was 9.1°C, the average frost-free period was 171 days and the mean annual precipitation was 584 mm. Rain falls mainly between June and September. Soil pH value is about 8.3, and the content of CaCO3 is 10.5%. The soil is a Calcarid Regosol according to the FAO/UNESCO classification system. The OC and N contents of the soils at the start of the experiment were 6.09 and 0.8 g kg-1, respectively. Soil loss due to erosion from water and wind is very low.

### Experimental design and soil sampling

The experiment was conducted in plots of 10.3 × 6.5 m with three replicates. Routine agriculture management practices were used in this region. Fertilizers were applied before seeding on soil, and plowed twice about 20 cm. In this study, three cropping systems were studied: continuously cropped alfalfa (*Medicago sativa*), continuously cropped winter wheat (*Triticum aestivum* L.), pea (*Pisum sativum* L.)-winter wheat-winter wheat-millet (*Panicum miliaceum* L.) rotation system (legume-grain rotation system). A fallow treatment, in which no crops were planted but which received the same practice of tillage as the cropping treatments, was used as a control.

For the continuously cropped alfalfa system, the alfalfa was sown in rows 50 cm apart in 1984 and was cut and removed from the plot twice each year. The land in the alfalfa plots was tilled with a moldboard plough to a depth of ~20 cm in mid-April of each year to minimize the effects of tillage among the cropping systems. Winter wheat was sown in mid-September and harvested in late June, pea was sown in mid-March and harvested in early July and millet was sown in early July and harvested in early October. Prior to seeding, the land was ploughed with a moldboard plough to a depth ~20 cm. Wheat and pea were sown in rows 25 cm apart. Millet was broadcast-sown. The crops were harvested at ground-level and removed from the plots. No crop was planted in the fallow plot. The land in the fallow plot was tilled in June of each year. The weeds in each plot were removed by hand, also including the fallow plot.

Previous studies have indicated that the stocks and distributions of soil OC and N in the fallow treatment did not change significantly during the experimental period [19–20]. Kaber et al found that the management of tillage had limited influence on the fractions of organic matter in the surface layer of silty soils under rotations of cereals with root crops [21]. We therefore assume that tillage had the same effects on the three cropping systems and the fallow treatment, and any changes in the distributions of aggregates and aggregate-associated OC and N were due to the cropping systems.

The soils and density fractions were treated with HCl to remove the carbonates before analysis. We measured soil bulk density in September 2011 at the 0–20 and 20–40 cm depths of each plot using a stainless-steel cutting ring 5.0 cm in length and 5.0 cm in diameter. Five random soil samples were collected from both depths in each plot with a tube auger 5 cm in diameter. Visible pieces of organic material were removed. The moist samples were air-dried in the laboratory and prepared for the analyses of soil aggregates and OC and N contents.

### Laboratory and statistical analyses

Four size classes of aggregates were separated by wet-sieving through 2, 0.25 and 0.053 mm sieves following the procedures described by Cambardella and Elliott [22]. The size-class samples were dried at 50–60°C, weighed and stored at room temperature. A subsample of air-dried, undisturbed soil from each plot was ground to pass through a 0.25-mm sieve for the measurement of OC and N in total soils. Soil OC and N in total soils and aggregates were analyzed using a C/N/H/S-analyzer (Vario ElementarIII, Germany).

Soil OC and N stocks (kg m^-2^) in bulk soils were calculated as:

$$\text{Soil OC }(\text{or N})\text{ stocks}=\frac{D\times \text{ρb}\times OC\;(or\;N)}{100}$$ (1)

where D is the thickness (cm) of the soil layer, ρb is the bulk density (g cm^-3^) and OC (or N) is the OC (or N) content (g kg^-1^) at the 0–20 or 20–40 cm soil depths. Stocks of OC (or N) (g m^-2^) in each size class of the 0–20 or 20–40 cm depths were calculated as:

$${\text{Stocks of OC }(\text{or N})}_{\text{i}}=\frac{D\times \text{ρb}\times w_{i}\times OC\;{\left(or\;N\right)}_{i}}{10}$$ (2)

where OC (or N)_*i*_ is the OC (or N) content of the *i*th aggregate size class (g kg^-1^ aggregates).

A two-way analysis of variance was conducted to test the effects of cropping system and soil depth on the distribution of soil aggregates, mean weight diameter, mean geometric diameter, OC and N contents and stocks in total soils and aggregates and the C/N ratio in total soils and aggregates. Linear regression analysis was used to examine the relationships between OC stocks in total soils and aggregates and between changes in OC stocks in total soils and aggregates. The variance and regression analyses were conducted using JMP version 10 software.

## Results

### The effects on bulk density and aggregate distribution

**Fig 1:**
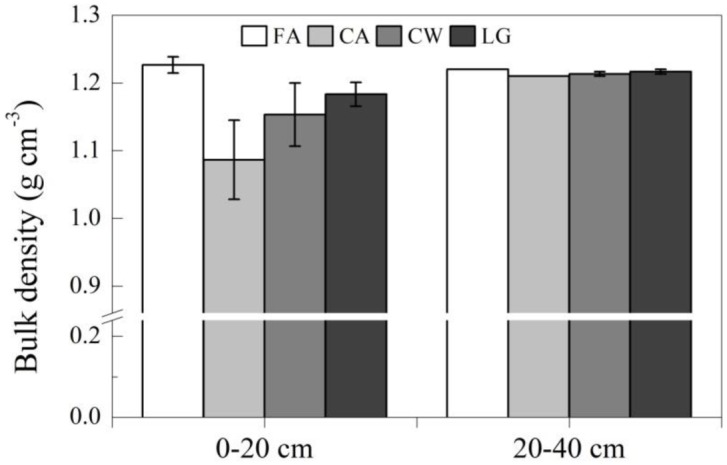
The effects of cropping systems on soil bulk density at the 0–20 and 20–40 cm depths. FA: Fallow treatment, CA: Continuously cropped alfalfa system; CW: continuously cropped winter wheat system; LG: Legume-grain rotation system.

Across all cropping systems, bulk density was 5% lower at the 0–20 cm depth than at the 20–40 cm depth (*P* = 0.0302). The continuous alfalfa system had the largest difference in bulk density between the two soil depths, and the fallow plot had the smallest difference. Compared with the fallow treatment, 27 years of cropping significantly decreased bulk density at the 0–20 cm depth (*P* = 0.0299) but did not affect bulk density at the 20–40 cm depth. The continuous alfalfa system had the largest decrease in bulk density (12%) (Fig 1).

**Fig 2:**
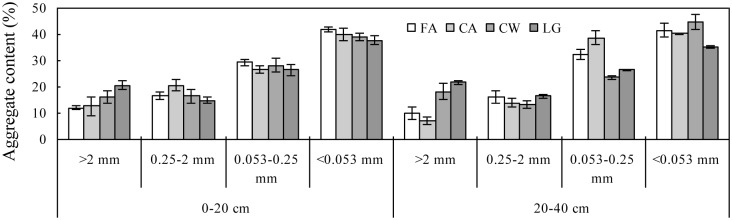
The effects of cropping systems on the distribution of aggregate size classes at the 0–20 and 20–40 cm depths. FA: Fallow treatment, CA: Continuously cropped alfalfa system; CW: continuously cropped winter wheat system; LG: Legume-grain rotation system.

The <0.053 mm size class accounted for most of the total soil mass (40%) across both depths, and the >2 and 0.25–2 mm size classes accounted for the least (16 and 17%, respectively). We did not observe a significant difference in size classes between the two soil depths across the cropping systems (Fig 2). The effect of cropping system on the distribution of aggregates varied with size class and soil depth (Fig 2). At the 0–20 cm depth, the amount of each size class was not affected by the cropping system (*P*>0.05). At the 20–40 cm depth, the amount of the >2 mm size class significantly increased in the continuous wheat and legume-grain rotation systems (by 82 and 117%, respectively) but decreased in the continuous alfalfa system (30%) (*P* = 0.031). The amount of the 0.053–0.25 mm size class significantly increased in the continuous alfalfa system (19%) but decreased in the continuous wheat and legume-grain rotation systems (by 27 and 19%, respectively) (*P* = 0.0010). The amounts of the 0.25–2 and <0.053 mm size classes were not affected by the cropping systems (*P*>0.05).

### The effects on OC and N in total soils

**Fig 3:**
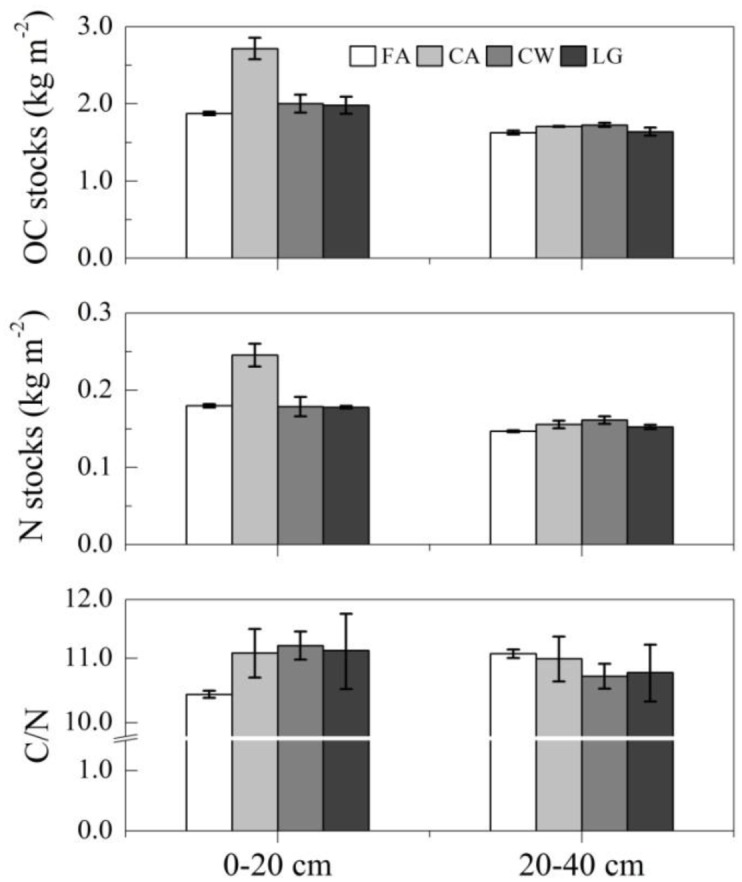
The effects of cropping systems on the OC and N stocks and C/N ratios in total soils at the 0–20 and 20–40 cm depths. FA: Fallow treatment, CA: Continuously cropped alfalfa system; CW: continuously cropped winter wheat system; LG: Legume-grain rotation system.

The stocks of OC and N across all cropping systems were 30 and 27% higher, respectively, at the 0–20 cm depth than at the 20–40 cm depth (*P* = 0.0004). Within the cropping systems, the stocks of OC and N were significantly higher at the 0–20 cm depth than at the 20–40 cm depth in the fallow treatment (15 and 22%, respectively), continuous alfalfa system (59 and 58%, respectively) and legume-grain rotation system (21 and 27%, respectively) (Fig 3).

Long-term cropping led to the recovery of OC and N in the soils (Fig 3). The cropping increased the stocks of OC and N in total soils at mean rates of 13.2 g OC m^-2^ yr^-1^ and 0.8 g N m^-2^ yr^-1^ at the 0–20 cm depth and of 2.4 g OC m^-2^ yr^-1^ and 0.4 g N m^-2^ yr^-1^ at the 20–40 cm depth. The continuous alfalfa system had the largest increases at the 0–20 cm depth. The stocks of OC and N in this system increased by 45 and 36%, respectively, (with recovery rates of 31.1 OC m^-2^ yr^-1^ and 2.4 g N m^-2^ yr^-1^) at the 0–20 cm depth and by 5 and 6%, respectively, (with recovery rates of 3.0 OC m^-2^ yr^-1^ and 0.03 g N m^-2^ yr^-1^) at the 20–40 cm depth. For the continuous wheat and legume-grain rotation systems, the stocks of OC increased by 6–7% at the 0–20 cm depth but decreased by 1% at the 20–40 cm depth. The stocks of N increased by 1–6 and 4–10% at the two depths, respectively. The C/N ratio or interaction was not affected by cropping system or soil depth (Fig 3).

### The effects on OC and N associated with aggregates

**Fig 4:**
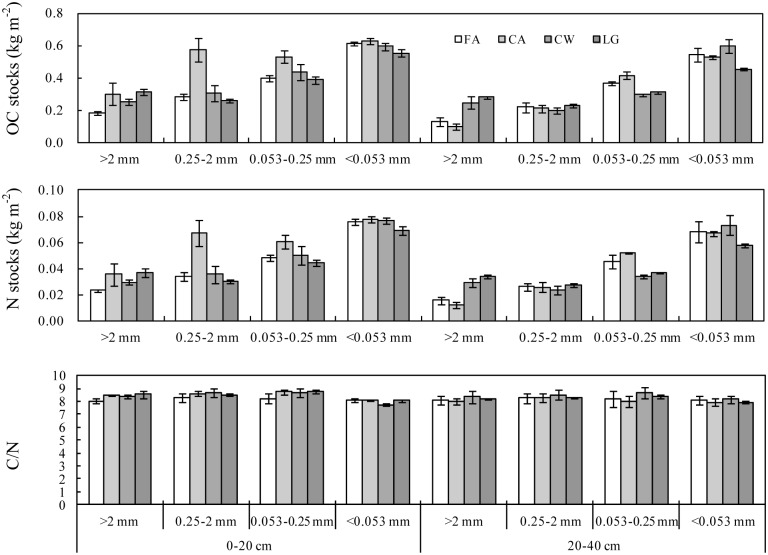
The effects of cropping systems on OC and N stocks and C/N ratios in aggregate size classes at the 0–20 and 20–40 cm depths. FA: Fallow treatment, CA: Continuously cropped alfalfa system; CW: continuously cropped winter wheat system; LG: Legume-grain rotation system.

The OC and N stocks in the aggregates varied significantly with soil depth, cropping system and their interactions (Table 1). Averaged across the cropping systems, the OC and N stocks associated with each size class were 12–67 and 13–63% higher, respectively, at the 0–20 cm depth than at the 20–40 cm depth (*P*<0.05). The stocks of OC and N in the <0.053 mm size class were 1.4–2.3 and 1.5–2.4 times higher, respectively, than the stocks in the other size classes (*P*<0.01) (Fig 4). Averaged across the 0–20 and 20–40 cm depths, 27 years of cropping increased the OC stocks in the >2, 0.25–2 and 0.053–0.25 mm size classes at mean rates of 3.37, 1.74 and 0.55 g m^-2^ yr^-1^, respectively, but decreased the OC stocks in the <0.053 mm size class at a mean rate of 0.66 g m^-2^ yr^-1^. The 27 years of cropping increased the N stocks in the <2 and 0.25–2 mm size classes at mean rates of 0.38 and 0.17 g m^-2^ yr^-1^, respectively, but decreased the N stocks in the 0.053–0.25 and <0.053 mm size classes at mean rates of 0.02 and 0.08 g m^-2^ yr^-1^, respectively.

**Table 1:** Analysis of variance results for all the variables.

	Cropping system		Soil depth		Interaction	
	F	P	F	P	F	P
Bulk density	2.62	0.0861	7.27	0.0159	1.97	0.1599
>2 mm size class content	12.24	0.0002	0.54	0.4724	1.34	0.2958
0.25–2 mm size class content	0.63	0.6034	3.23	0.0913	2.06	0.1457
0.053–0.25 mm size class content	6.91	0.0034	4.17	0.0580	7.83	0.0019
<0.053 mm size class content	3.68	0.0344	0.38	0.5488	1.87	0.1761
OC stored in total soils	13.91	0.0001	71.37	<0.0001	10.69	0.0004
N stored in total soils	10.92	0.0004	62.15	<0.0001	9.73	0.0007
C/N in total soils	0.25	0.8631	0.10	0.7610	1.06	0.3949
OC stocks in >2 mm size class	6.99	0.0032	10.81	0.0046	3.83	0.0304
OC stocks in 0.25–2 mm size class	8.03	0.0017	30.56	<0.0001	8.69	0.0012
OC stocks in 0.053–0.25 mm size class	8.75	0.0011	22.94	0.0002	1.56	0.2376
OC stocks in <0.053 mm size class	4.91	0.0132	13.06	0.0023	1.69	0.2091
N stocks in >2 mm size class	7.14	0.0029	10.58	0.0050	3.83	0.0304
N stocks in 0.25–2 mm size class	5.93	0.0064	21.40	0.0003	6.18	0.0054
N stocks in 0.053–0.25 mm size class	7.16	0.0029	10.91	0.0045	1.11	0.3723
N stocks in <0.053 mm size class	2.83	0.0714	7.33	0.0155	0.35	0.7867
C/N in >2 mm size class	0.57	0.6431	1.17	0.2946	0.54	0.6609
C/N in 0.25–2 mm size class	0.48	0.6998	0.80	0.3835	0.09	0.9629
C/N in 0.053–0.25 mm size class	0.72	0.5524	1.17	0.2957	0.43	0.7352
C/N in <0.053 mm size class	0.17	0.9177	0.07	0.7986	0.95	0.4414

The changes in the stocks of OC and N associated with the aggregates varied with cropping system and soil depth. The legume-grain rotation system decreased the OC and N stocks in the <2 mm size class (2–9 and 8–12%, respectively) but increased the OC and N stocks in the >2 mm size class (71 and 61%, respectively) at the 0–20 cm depth. At the 20–40 cm depth, the legume-grain rotation system decreased the stocks of OC and N in the 0.053–0.25 and <0.053 mm size classes (15 and 16% for OC and 18 and 16% for N, respectively) but increased the OC and N stocks in the >2 and 0.25–2 mm size classes (118 and 6% for OC and 120 and 5% for N, respectively) (Fig 4).

The continuous alfalfa system led to larger increases in OC (34–105%) and N (26–97%) stocks in the >0.053 mm size class but to smaller increases in the <0.053 mm size class at the 0–20 cm depth. At the 20–40 cm depth, the OC and N stocks in the 0.053–0.25 mm size class increased by 14 and 15%, respectively, and the OC and N stocks in the other size classes decreased by 2–26 and 2–23%, respectively (Fig 4).

The continuous wheat system led to 9–35 and 4–28% increases in the OC and N stocks, respectively, in the >0.053 mm size class but to no changes in the <0.053 mm size class at the 0–20 cm depth. At the 20–40 cm depth, this system led to 10 and 12% decreases in the OC and N stocks in the 0.25–2 mm size class and to 19 and 25% decreases in the 0.053–0.25 mm size class, respectively, but to 93 and 87% increases in the >2 mm size class and to 10 and 8% increases in the <0.053 mm size class, respectively (Fig 4).

### The effects on C/N ratios in the aggregates

The C/N ratios averaged across all cropping systems were relatively higher in the >0.053 mm size class than the <0.053 mm size class at both depths. For example, the average C/N ratios in the >2, 0.25–2 and 0.053–0.25 mm size classes were 3–7% higher at the 0–20 cm depth and 1–6% higher at the 20–40 cm depth than in the <0.053 mm size class (Fig 4).

The effect of long-term cropping on the C/N ratio in each size class varied with soil depth. At the 0–20 cm depth, 27 years of cropping increased the C/N ratio in the >0.053 mm size class but decreased it in the <0.053 mm size class. At the 20–40 cm depth, the continuous wheat and legume-grain rotation systems increased the C/N ratio in most aggregates, while the continuous alfalfa system decreased the C/N ratio in most aggregates (Fig 4).

### The contribution of the changes in aggregate-associated OC and N to the recovery of OC and N in total soils

**Fig 5:**
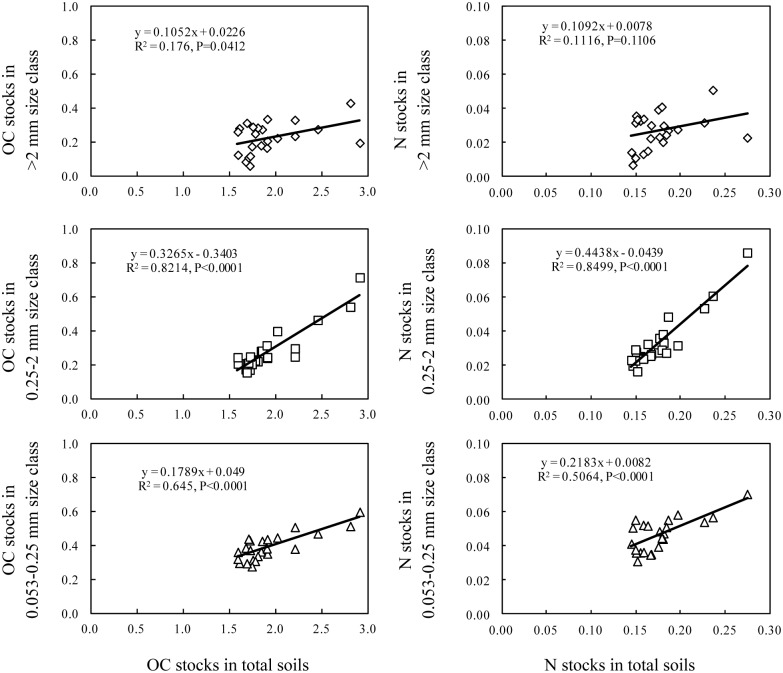
The relationships between OC and N stocks in total soils and OC and N stocks in aggregate size classes. The units for OC and N stocks in total soils and aggregates are kg m^-2^.

**Fig 6:**
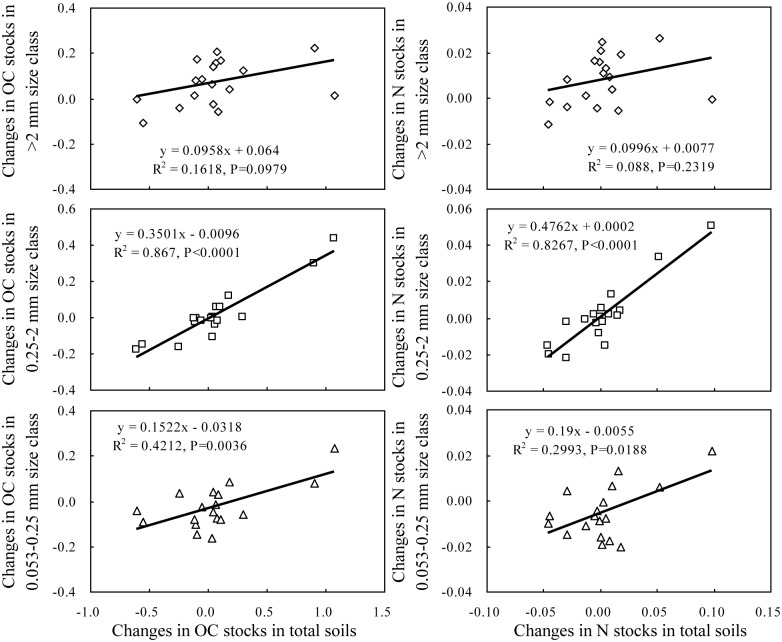
The relationships between changes in OC and N stocks in total soils and changes in OC and N stocks in aggregate size classes. The units for the changes in OC and N stocks in both total soils and aggregates are kg m^-2^.

The changes in the OC and N stocks in total soils after 27 years of cropping were mainly due to the changes in the OC and N stocks associated with the aggregates >0.053 mm. The OC and N stored in total soils were significantly correlated with the OC and N stored in the >2, 0.25–2 and 0.053–0.25 mm size classes (Fig 5). The changes in the OC and N stocks in total soils were also significantly correlated with the changes in the OC and N stocks in the >2, 0.25–2 and 0.053–0.25 mm size classes (Fig 6). Additionally, the OC and N stocks in the continuous alfalfa system in the >2, 0.25–2 and 0.053–0.25 mm size classes throughout the 0–40 cm depth increased by 84, 289 and 184 g OC m^-2^ and by 9, 33 and 19 g N m^-2^, respectively, which accounted for 99.5 and 98.7% of the total increases in OC and N, respectively. Similarly, the increases in the OC and N associated with the >0.25 mm size class accounted for 97 and 100% of the total increases in OC and N in the continuous wheat system and for 100 and 100% of the total increases in the legume-grain rotation system, respectively.

## Discussion

Our results showed that the cropping system did not affect the distribution of soil aggregates at the 0–20 cm depth but significantly changed the distribution at the 20–40 cm depth. These results were expected because each system, including the fallow treatment, received the same tillage practice, which occurred at the 0–20 cm depth. The aggregate distribution changed significantly at the 20–40 cm depth mainly due to the activities of the plant roots in the deeper soils. Plant roots can significantly accelerate the aggregation of soil particles [23]. The effects on the aggregate distribution varied with cropping system. Alfalfa has tap roots, and the development of coarse roots in soils leads to increases of macroaggregates (>0.25 mm). Wheat and millet have fibrous roots, and the development of fine roots in soils leads to increases of microaggregates (0.053–0.25 mm). More roots were distributed at the 0–20 cm depth than at the 20–40 cm depth in the cropping systems, but the breakdown of aggregates induced by tillage is greater than the aggregation of particles induced by roots [12–15].

Intense tillage and the conversion of natural vegetation to cropland can lead to the loss of soil OC and N [3, 11–13, 24]. Changes in soil OC and N after long-term cropping, however, have not been well studied but would provide essential information for understanding the potentials of C sequestration in agricultural soils. Our results demonstrated the recovery of OC and N in semiarid highland agricultural soils. These results are consistent with other findings that OC and N accumulate in both untilled and tilled soil [17, 25, 26]. For example, Somnrero and de Benito observed significant increases in soil OC after ten years of conservative tillage and crop rotation in a semiarid area of Spain [26]. Norton et al reported that the wheat-fallow system and a conservative system of tillage increased soil OC content by 66 and 73% [17], respectively, compared with the traditional system, in a semiarid in southeastern Wyoming. The recovery of soil OC and N after the long-term planting of crops is mainly attributed to the accumulation of root biomass in the soils, because the aboveground biomass and crop litter are generally removed from the land. Roots play a primary role in the dynamics of soil OC and N in farmland, grassland and forest and after changes in land use [24, 27, 28]. The leaching of OC and N from crop canopies may be another source of soil OC and N in cropping systems [29].

In addition to the direct input of OC and N to soils, the roots in cropping systems provide a C source for microbial activity, which, together with the root biomass and exudates, induces the binding of residues and soil particles into aggregates [24, 30]. In our study, the amounts of macro- and/or microaggregates were significantly increased by the cropping systems compared with the fallow treatment (Fig 2), which enhanced the physical protection of the initial and newly input OC and N from mineralization and thus decreased the loss of soil OC and N [31].

Additionally, the crop cover may have helped to reduce the loss of soil OC and N in the cropping systems relative to the fallow treatment. The interception of raindrops by crop canopies leads to less destruction of soil structure and less loss of soil OC and N relative to fallow treatments [32]. Crop covers also decrease the variability of soil temperature and water in cropping systems compared with fallow treatments [33], which decreases the mineralization of soil OC and N and thus their loss from soils. Highly variable soil temperatures increase the mineralization of soil organic matter and the release of CO_2_ and N_2_O from soils [34]. In our study, soil OC and N contents in the fallow treatment decreased by 1 and 5%, respectively, after 27 years. Although not directly verified, the decreases of initial soil OC and N in cropping systems should be lower than those in fallow treatments. We therefore assume that the recovery of soil OC and N in the cropping systems was mainly due to the increased accumulation of newly input OC and N and to the decreased loss of the initial OC and N.

The continuous alfalfa system had the highest recovery rates of OC and N due to its large biomass. The biomass of this system averaged over the 27 years was 10 and 17% higher than those of the continuous wheat and legume-grain rotation systems, respectively (data not shown). Additionally, the improvement of soil structure in rotation systems with legumes, by increasing the amounts of aggregates, can provide more physical protection to the soil OC and N. For example, Hajabbasi and Fallahzade observed higher stabilities of aggregates and OC in a rotation system that included a legume (alfalfa and wheat) than in a rotation system without a legume (wheat-fallow-barley) in arid sandy soils in Iran [35].

In addition to high biomass and the improvement of soil structure, the fixation of N by alfalfa also contributes to higher OC and N recovery rates, because the accumulation of N enhances the sequestration of OC in soils [36, 37]. Similarly, the lower amounts of biomass in the continuous wheat system in our study led to lower recovery rates of OC and N in the soil. These results were consistent with the findings that legume plots had higher levels of soil OC than cereal plots and that higher plant biomasses often led to more soil OC sequestration [26, 38, 39]. Huggins et al, however, reported that continuous corn and corn-soybean systems had relatively higher recovery rates of soil OC (0.45–3.35 and 0.38–2.53 Mg C ha^-1^ yr^-1^, respectively) and that a continuous soybean system had a relatively lower recovery rate (0.02–1.29 Mg C ha^-1^ yr^-1^) throughout the 0–45 cm depth, because the biomass was larger for corn than for soybean [40].

Our results showed that the recovery of OC and N mainly occurred in the >0.053 mm size class and varied with cropping system. The recovery in the continuous alfalfa system mainly occurred in the >2, 0.25–2 and 0.053–0.25 mm size classes (557 g OC m^-2^ and 61 g N m^-2^), and the recovery in the continuous wheat and rotation systems mainly occurred in the >2 mm size class. The stabilities of the recovered OC and N in the aggregates, however, were determined by the size class. Tillage often breaks up aggregates and decreases their stability and thus decreases their ability to physically protect soil organic matter [11, 41]. Additionally, the C/N ratios averaged across all cropping systems throughout the 0–40 cm depth were relatively higher in the 0.25–2 and 0.053–0.25 mm size classes than in the >2 mm size class (Fig 4). Lower C/N ratios favor the decomposition of organic materials [11, 42] and thus decrease the stability of organic matter in macroaggregates. Organic matter associated with macroaggregates is also assumed to be more susceptible to loss due to mineralization compared with organic matter associated with microaggregates [11, 43]. For example, the turnover rate of organic matter can increase as aggregate size increases and mean residence time decreases [11, 43]. The sequestrated OC and N in our study were thus relatively more stable in the continuous alfalfa system than in the continuous wheat and legume-grain rotation systems.

We have shown that various cropping systems have the potential to recover soil OC and N due to the accumulation of above- and belowground biomass. We therefore assume that any practices that can increase the biomass, particularly belowground biomass, such as irrigation and fertilization, have the potential to enhance the recovery of soil OC and N in semiarid agroecosystems.

## Conclusions

We demonstrated that long-term cropping led to the recovery of OC and N in soils of a semiarid highland agroecosystem at mean recovery rates of 15.6 g OC m^-2^ yr^-1^ and 1.2 g N m^-2^ yr^-1^ throughout the 0–40 cm depth. The continuous alfalfa system had the highest recovery. Recovery mainly occurred in the >0.053 mm aggregate size class, which varied with cropping system. The continuous alfalfa system led to the accumulation of OC and N in the 0.25–2 and 0.053–0.25 mm size classes, and the continuous wheat and rotation systems led to the accumulation of OC and N in the >2 mm size class. The accumulated OC and N were relatively more stable in the continuous alfalfa system than in the continuous wheat and rotation systems.
